# Severity of Nausea and Vomiting in Singleton and Twin Pregnancies in Relation to Fetal Sex: The Japan Environment and Children’s Study (JECS)

**DOI:** 10.2188/jea.JE20180059

**Published:** 2019-09-05

**Authors:** Naomi Mitsuda, Masamitsu Eitoku, Nagamasa Maeda, Mikiya Fujieda, Narufumi Suganuma

**Affiliations:** 1 Department of Environmental Medicine, Kochi Medical School, Kochi University, Kochi, Japan; 2 Department of Obstetrics and Gynecology, Kochi Medical School, Kochi University, Kochi, Japan; 3 Department of Pediatrics, Kochi Medical School, Kochi University, Kochi, Japan

**Keywords:** nausea and vomiting during pregnancy, fetal sex, twin, multiple birth, JECS

## Abstract

**Background:**

Some studies have indicated that female birth and multiple births were risk factors for nausea and vomiting during pregnancy (NVP). The results, however, were conflicting. Our study was conducted to evaluate the association of maternal NVP with fetal sex in singleton and twin pregnancies.

**Methods:**

We used the data set from a birth cohort study, the Japan Environment and Children’s Study (JECS). In the self-administered questionnaire, participants were asked whether they experienced NVP prior to 12 gestational weeks. Main outcome measures were the presence of NVP and severity of NVP. We estimated the association of fetal sex and birth plurality with NVP using logistic regression analysis, followed by interaction analysis.

**Results:**

Of 91,666 women, 75,828 (82.7%) experienced at least some symptoms of NVP and 10,159 (11.1%) experienced severe NVP. Women with female pregnancies and twin pregnancies had higher odds for the presence of NVP and severe NVP compared to women with male pregnancies and singleton pregnancies, respectively. Moreover, of mothers with twin pregnancies, higher odds for the presence of NVP and severe NVP were reported when one or both infants were female, compared to those in which both infants were male. There was no significant interaction between fetal sex and birth plurality.

**Conclusions:**

Female sex birth and multiple births are risk factors for the presence of NVP, and especially for severe NVP without interaction. These findings suggest that a factor abundant in the female fetus associates with the severity of NVP.

## INTRODUCTION

Nausea and vomiting during pregnancy (NVP) is one of the most common clinical conditions women experience in the first trimester of pregnancy. It is estimated that 70–80% of pregnant women develop at least some symptoms of NVP.^1^^,^^2^ An extreme form of NVP, accompanied by weight loss, dehydration, and electrolyte and metabolic disorders, is referred to as hyperemesis gravidarum (HG), affecting 0.3–2% of all pregnancies.^3^

Various genetic, metabolic and endocrine factors have been considered as relevant to the mechanisms of NVP or HG, and among these, human chorionic gonadotropin (hCG) is thought to be one of the most dominant. However, a clear etiopathogenesis of NVP has not yet been identified.

Some risk factors for NVP or HG have also been described. Maternal genetic factors appear to serve as primary risk factors for NVP or HG.^4^^,^^5^ Furthermore, NVP is considered to be more common in younger women, women with less than 12 years of education, non-smokers, obese women, and women with multiple gestation.^6^^–^^8^ With regard to fetal sex, many studies have reported an association between HG and fetal sex, and almost all of these studies confirmed female sex birth as a risk factor for HG.^9^^–^^12^ A few studies examined the joint effect of twinning and fetal sex on HG and showed that the presence of at least one female in the twin pair was associated with HG.^10^^,^^13^^,^^14^ On the other hand, a few studies reported an association between NVP and fetal sex. Some of these studies found that female sex birth was a risk factor for NVP.^6^^,^^13^ However, the results were conflicting and there is no study evaluating the association of NVP with both fetal sex and birth plurality.^14^

Based on these conflicting results of past studies, it is necessary to assess the effect of fetal sex and birth plurality on the NVP in a large cohort study; especially to assess the joint effect or the interaction between these factors on NVP, studies with large sample size are needed. Even though these fetal sex and birth plurality factors have been shown to be associated with NVP, the findings may not directly lead to prevention or treatment of NVP. However, the findings may be predictive factors of severity of NVP and also may reduce the anxieties of pregnant women who are conscious of the association between severity of NVP and poor birth outcome. So, in this study, we evaluated NVP in twin and singleton pregnancies in relation to fetal sex using data from the Japan Environment and Children’s Study (JECS).

## METHODS

### Study design

We retrospectively analyzed the data set from the JECS. The JECS is a birth cohort study undertaken to elucidate the influence of chemical exposure during the fetal period and early childhood on children’s health, with follow-up until age 13. The protocol and baseline data of this study is available elsewhere.^15^^,^^16^ The Ministry of Environment organized a national research group headed by the National Institute of Environmental Studies in collaboration with the National Center for Child Health and Development and 15 regional centers.

For the JECS, pregnant women were recruited between January 2011 and March 2014. Eligibility criteria for participants (expectant mothers) were as follows: 1) residing in the study areas at the time of recruitment and enrolled with collaborating health care providers; 2) expected delivery date after August 1, 2011; and 3) capable of comprehending the Japanese language and completing the self-administered questionnaire. Details of the JECS project have been described in a previous article.^16^

The JECS protocol was approved by the Institutional Review Board on Epidemiological Studies of the Ministry of the Environment and the ethics committees of all participating institutions. The JECS was conducted in accordance with the Declaration of Helsinki and other internationally valid regulations and guidelines and with written informed consent from all participants.

With regard to exposure measurement, lifestyle and other background information was collected using a self-administered questionnaire distributed to participating pregnant women at the first trimester (M-T1) and the second/third trimester in pregnancy (M-T2). Medical histories of past and present pregnancies and participants’ and their offspring’s physical status were transcribed from an obstetrician’s medical chart at registration (Dr-T1) and at delivery (Dr-0 m). Analyses of this study were based on M-T2, Dr-T1, and Dr-0 m.

### Sample selection

**Figure 1.**
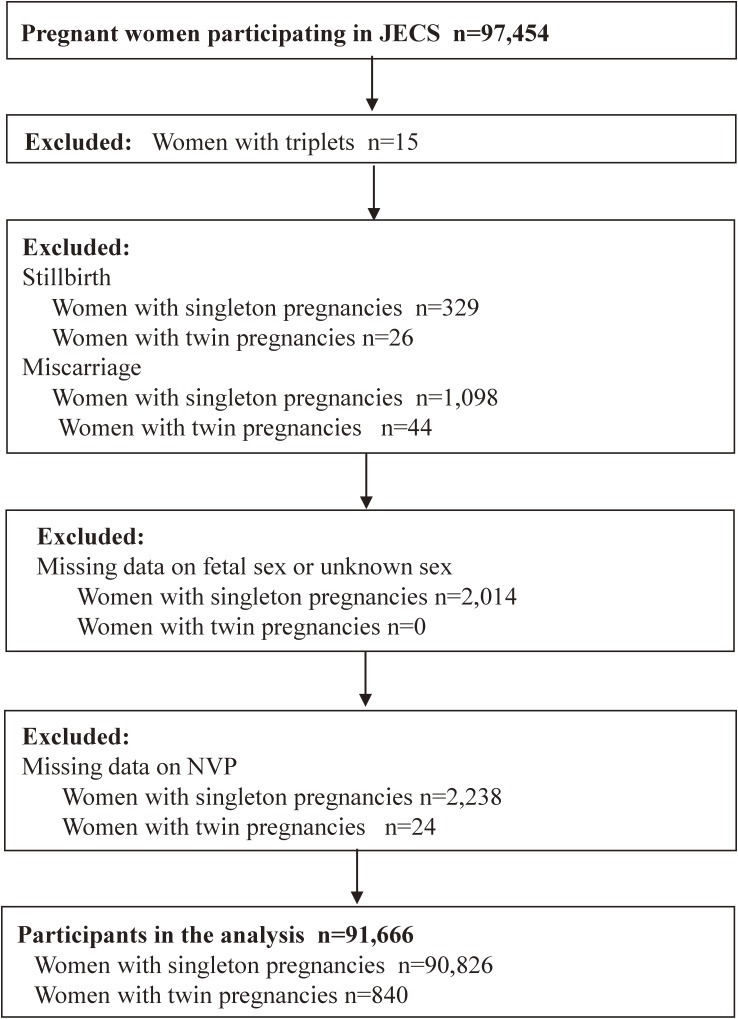
Flow chart for selection of participants from JECS

The present study was based on the “jecs-ag-20160424”, which was released in June, 2016. The data set included 104,102 fetal records and 97,454 women’s records. Of 97,454 women, 4,858 women participated two or three times in the JECS, and we included their first records only in this analysis. We excluded women with triplets (*n* = 15), cases of stillbirth (329 women with singleton births and 26 women with twin births) or cases with miscarriage (1,098 women with singleton and 44 with twin miscarriage). We also excluded cases with missing data on the sex of offspring or undetermined sex of offspring (2,014 women with singleton births), and cases with missing data on NVP (2,238 women with singleton and 24 with twin births). In total, 91,666 women (90,826 with singleton and 840 with twin births) were included the final study sample (Figure 1).

### Variables

Information on NVP, maternal education, and maternal smoking habits during pregnancy were obtained from M-T2. In M-T2, participants were asked whether they experienced NVP prior to 12 gestational weeks, and responses were categorized as 1) did not experience NVP; 2) nausea only; 3) experienced NVP but could have meals; or 4) experienced NVP and could not have meals.

Information on parity, maternal height, and pre-pregnancy weight were obtained from Dr-T1. Maternal age, birth plurality, and birth outcomes were obtained from Dr-0m and sex of offspring was obtained from revised data. The women with singleton pregnancies were divided into two groups according to fetal sex. The women with twins were split into three groups according to fetal sex combinations: male-male, male-female, and female-female.

Maternal age was categorized into six groups: younger than 20 years, 20–24 years, 25–29 years, 30–34 years, 35–39 years, and 40 years and older. Pre-pregnancy body mass index (BMI) was calculated from the information on pre-pregnancy height and weight and categorized into three groups: underweight (<18.5 kg/m^2^), normal (18.5–24.9 kg/m^2^) and overweight (≥25 kg/m^2^). Data on parity were classified into primipara and multipara. A proxy for socioeconomic status, maternal length of education, was categorized into ≤12 years and >12 years. Maternal smoking habits were categorized into smoking during pregnancy and others.

### Statistical analyses

The study population was divided into four groups depending on the answers to the questionnaire for the symptoms of NVP as follows: those who did not experience NVP (no NVP); those who experienced nausea only (nausea only); those who experienced NVP but could have meals (moderate NVP); and those who experienced NVP and could not have meals (severe NVP). Maternal characteristics were compared among the four NVP groups. All categorical variables were compared employing a chi-squared test and effect sizes were assessed using Cramer’s V. Tests for trend were also performed by including the NVP categories as continuous variables.

Main study outcomes were the presence of NVP and severity of NVP. To evaluate the presence or absence of NVP, four types of answer for NVP were dichotomized into “no NVP/nausea only” and “moderate NVP/severe NVP”, respectively. Similarly, in order to evaluate the severity of NVP, four types of answer for NVP were dichotomized into “severe NVP” and others. Logistic regression analysis was performed to estimate the association of the presence of NVP and the severity of NVP with fetal sex in singleton and twin pregnancies. Results are presented as crude odds ratios (cOR), adjusted odds ratios (aOR), and mean differences with 95% confidence intervals (CIs). Maternal age, pre-pregnancy BMI, parity, smoking during pregnancy, and maternal education were used as confounders for calculating aORs.

To examine whether the effects of the fetal sex on NVP differed in the two types of birth plurality, we assessed the interaction between the number of female fetuses and birth plurality by centering on the number of female fetuses to avoid multicollinearity. All analyses were performed using Stata 13.1 (Stata Corp, College Station, TX, USA).

## RESULTS

Maternal characteristics according to NVP status are presented in Table 1. The 91,666 women were categorized into “no NVP” (*n* = 15,838; 17.3%), “nausea only” (*n* = 39,202; 42.8%), “moderate NVP” (*n* = 26,467; 28.9%), and “severe NVP” (*n* = 10,159; 11.1%). Higher prevalence of “no NVP” was seen among women of advanced age, those with low pre-pregnancy BMI, primipara, those with lower education, and those who smoked during pregnancy. The prevalence of twins in all participants was 0.9%, whereas rates were 0.8% and 1.4% in women without NVP and in women with severe NVP, respectively. All tests for trend for the associations between NVP and these factors, except for maternal education, were significant, although the effect sizes were small (Table 1). Women with singleton pregnancies were younger than women with twins, and the percentage of multipara was higher among singleton pregnancies (eTable 1).

**Table 1.** Maternal characteristics according to NVP status

	Total	No NVP	Nausea only	Moderate NVP	Severe NVP	*P* value	*P*-for trend	Cramer’s V
	*n* = 91,666	*n* = 15,838	*n* = 39,202	*n* = 26,467	*n* = 10,159			
	*N* (%)	*N* (%)	*N* (%)	*N* (%)	*N* (%)			
**Maternal age, years**								
<20	800	190 (1.2)	247 (0.6)	254 (1.0)	109 (1.1)	<0.001	<0.001	0.051
20–24	8,169	1,583 (10.0)	2,839 (7.2)	2,646 (10.0)	1,101 (10.8)			
25–29	25,202	4,276 (27.0)	10,196 (26.0)	7,686 (29.0)	3,044 (30.0)			
30–34	32,410	5,164 (32.6)	14,238 (36.3)	9,471 (35.8)	3,537 (34.8)			
35–39	20,827	3,710 (23.4)	9,642 (24.6)	5,453 (20.6)	2,022 (19.9)			
≥40	4,254	912 (5.8)	2,040 (5.2)	956 (3.6)	346 (3.4)			
Missing^a^	4	3	0	1	0			
**Pre-pregnancy BMI, kg/m^2^**								
<18.5	14,846	2,739 (17.3)	6,511 (16.6)	4,010 (15.2)	1,586 (15.6)	<0.001	<0.001	0.028
18.5–24.9	67,048	11,602 (73.3)	28,772 (73.4)	19,411 (73.4)	7,263 (71.5)			
≥25	9,713	1,486 (9.4)	3,895 (9.9)	3,029 (11.5)	1,303 (12.8)			
Missing^a^	59	11	24	17	7			
**Parity**								
Primipara	38,153	8,303 (54.3)	15,532 (40.6)	10,141 (39.1)	4,177 (42.2)	<0.001	<0.001	0.108
Multipara	51,248	6,990 (45.7)	22,747 (59.4)	15,781 (60.9)	5,730 (57.8)			
Missing^a^	2,265	545	923	545	252			
**Education, years**								
≤12	33,012	6,059 (38.4)	13,361 (34.2)	9,791 (37.1)	3,801 (37.6)	<0.001	0.141	0.036
>12	58,295	9,711 (61.6)	25,700 (65.8)	16,568 (62.9)	6,316 (62.4)			
Missing^a^	359	68	141	108	42			
**Smoking during pregnancy**								
No	86,787	14,635 (93.3)	37,288 (95.9)	25,091 (95.6)	9,773 (97.0)	<0.001	<0.001	0.051
Yes	4,129	1,049 (6.7)	1,606 (4.1)	1,169 (4.5)	305 (3.0)			
Missing^a^	750	154	308	207	81			
**Number of fetuses**								
Singleton	90,826	15,714 (99.2)	38,888 (99.2)	26,205 (99.0)	10,019 (98.6)	<0.001	<0.001	0.019
Twin	840	124 (0.8)	314 (0.8)	262 (1.0)	140 (1.4)			

BMI, body mass index; NVP, nausea and vomiting in pregnancy.

Chi-squared test.

^a^Not included in percentage distribution.

The male-to-female sex ratio was 1.05 (95% CI, 1.04–1.07) in all singleton pregnancies, whereas it was 1.21 (95% CI, 1.17–1.25) and 0.85 (95% CI, 0.82–0.88) in singleton pregnancies among women without NVP and women with severe NVP, respectively (Table 2). In the logistic regression analysis, when compared to women with singleton pregnancies, women with twin pregnancies had significantly increased odds for severe NVP (aOR 1.61; 95% CI, 1.34–1.94) after adjustment for maternal age, parity, smoking during pregnancy, maternal education, and the presence of female fetuses. Women with twin pregnancies also had increased odds for the presence of NVP (aOR 1.41; 95% CI, 1.23–1.62) (Table 3). When compared to women with male singleton pregnancies, women with female singleton, male-male twin, male-female twin, and female-female twin pregnancies had significantly increased odds for the presence of NVP (aOR 1.15; 95% CI, 1.12–1.18, aOR 1.36; 95% CI, 1.08–1.70, aOR 1.68; 95% CI, 1.27–2.21, and aOR 1.65; 95% CI, 1.31–2.07, respectively) and increased odds for severe NVP (aOR 1.28; 95% CI, 1.23–1.34, aOR 1.27; 95% CI, 0.91–1.79, aOR 2.01; 95% CI, 1.40–2.89, and aOR 2.51; 95% CI, 1.90–3.32, respectively) (Table 4). There was no significant interaction between fetal sex and birth plurality on NVP (eTable 2).

**Table 2.** Fetal sex according to NVP status

	Total	No NVP	Nausea only	Moderate NVP	Severe NVP	*P* value	Cramer’s V
	*n* = 91,666	*n* = 15,838	*n* = 39,202	*n* = 26,467	*n* = 10,159		
	*N* (%)	*N* (%)	*N* (%)	*N* (%)	*N* (%)		
**Singleton (*n* = 90,826)**							
Male	46,581 (51.3)	8,610 (54.8)	20,130 (51.8)	13,244 (50.5)	4,597 (45.9)	<0.001	0.047
Female	44,245 (48.7)	7,104 (45.2)	18,758 (48.2)	12,961 (49.5)	5,422 (54.1)		
**Twin (*n* = 840)**							
Both male	322 (38.3)	56 (45.2)	122 (38.9)	106 (40.5)	38 (27.1)	<0.001	0.087
Male & female	213 (25.4)	29 (23.4)	79 (25.2)	69 (26.3)	36 (25.7)		
Both female	305 (36.3)	39 (31.5)	113 (36.0)	87 (33.2)	66 (47.1)		

NVP, nausea and vomiting in pregnancy.

Chi-squared test.

**Table 3.** Odds ratio of presence of NVP and severe NVP in relation to plurality

	Presence of NVP			Severe NVP		
	cOR (95% CI)	aOR^a^ (95% CI)	aOR^b^ (95% CI)	cOR (95% CI)	aOR^a^ (95% CI)	aOR^b^ (95% CI)
Singleton	1 (Reference)	1 (Reference)	1 (Reference)	1 (Reference)	1 (Reference)	1 (Reference)
Twin	1.38 (1.21–1.59)	1.44 (1.25–1.65)	1.41 (1.23–1.62)	1.61 (1.34–1.94)	1.66 (1.38–2.00)	1.61 (1.34–1.94)

aOR, adjusted odds ratio; CI, confidence interval; cOR, crude odds ratio; NVP, nausea and vomiting in pregnancy.

^a^Adjusted for maternal age, pre-pregnancy BMI, parity, smoking during pregnancy, and maternal education.

^b^Additional adjustment for the presence of female fetuses.

**Table 4.** Odds ratio for presence of NVP and severe NVP in relation to fetal sex and plurality

	Presence of NVP		Severe NVP	
	cOR (95% CI)	aOR (95% CI)	cOR (95% CI)	aOR (95% CI)
**Fetal sex and plurality^a^**				
Singleton, male	1 (Reference)	1 (Reference)	1 (Reference)	1 (Reference)
Singleton, female	1.15 (1.12–1.18)	1.15 (1.12–1.18)	1.28 (1.22–1.33)	1.28 (1.23–1.34)
Twin, both male	1.30 (1.05–1.62)	1.36 (1.08–1.70)	1.22 (0.87–1.72)	1.27 (0.91–1.79)
Twin, male & female	1.57 (1.20–2.05)	1.68 (1.27–2.21)	1.86 (1.30–2.66)	2.01 (1.40–2.89)
Twin, both female	1.62 (1.29–2.03)	1.65 (1.31–2.07)	2.52 (1.92–3.32)	2.51 (1.90–3.32)
**Maternal age, years^b^**				
<20	1 (Reference)	1 (Reference)	1 (Reference)	1 (Reference)
20–24	1.02 (0.88–1.18)	0.94 (0.81–1.10)	0.99 (0.80–1.22)	0.91 (0.73–1.14)
25–29	0.89 (0.77–1.03)	0.78 (0.67–0.90)	0.87 (0.71–1.07)	0.79 (0.64–0.98)
30–34	0.81 (0.70–0.93)	0.67 (0.58–0.78)	0.78 (0.63–0.95)	0.69 (0.56–0.86)
35–39	0.67 (0.58–0.78)	0.54 (0.47–0.63)	0.68 (0.55–0.84)	0.60 (0.48–0.75)
≥40	0.53 (0.46–0.62)	0.43 (0.36–0.50)	0.56 (0.45–0.71)	0.49 (0.38–0.62)
**Pre-pregnancy BMI, kg/m^2 c^**				
<18.5	0.92 (0.88–0.95)	0.88 (0.85–0.92)	0.98 (0.93–1.04)	0.95 (0.90–1.01)
18.5–24.9	1 (Reference)	1 (Reference)	1 (Reference)	1 (Reference)
≥25	1.22 (1.16–1.27)	1.23 (1.18–1.29)	1.28 (1.20–1.36)	1.29 (1.21–1.38)
**Parity^d^**				
Primipara	1 (Reference)	1 (Reference)	1 (Reference)	1 (Reference)
Multipara	1.20 (1.17–1.24)	1.31 (1.27–1.35)	1.02 (0.98–1.07)	1.10 (1.05–1.15)
**Education, years^e^**				
≤12	1 (Reference)	1 (Reference)	1 (Reference)	1 (Reference)
>12	0.92 (0.90–0.95)	1.00 (0.97–1.03)	0.93 (0.89–0.97)	0.98 (0.93–1.02)
**Smoking during pregnancy^f^**				
No	1 (Reference)	1 (Reference)	1 (Reference)	1 (Reference)
Yes	0.83 (0.77–0.88)	0.76 (0.71–0.81)	0.63 (0.56–0.71)	0.59 (0.52–0.66)

aOR, adjusted odds ratio; BMI, body mass index; CI, confidence interval; cOR, crude odds ratio; NVP, nausea and vomiting in pregnancy.

^a^Adjusted for maternal age, pre-pregnancy BMI, parity, education, and smoking during pregnancy.

^b^Adjusted for fetal sex and plurality, pre-pregnancy BMI, parity, education, and smoking during pregnancy.

^c^Adjusted for fetal sex and plurality, maternal age, parity, education, and smoking during pregnancy.

^d^Adjusted for fetal sex and plurality, maternal age, pre-pregnancy BMI, education, and smoking during pregnancy.

^e^Adjusted for fetal sex and plurality, maternal age, pre-pregnancy BMI, parity, and smoking during pregnancy.

^f^Adjusted for fetal sex and plurality, maternal age, pre-pregnancy BMI, parity, and education.

## DISCUSSION

Our results show that women with female singleton pregnancies have increased odds of both the presence of NVP and severe NVP compared to women with male singleton pregnancies. When compared to women with singleton pregnancies, women with twin pregnancies had significantly increased odds for the presence of NVP and increased odds for severe NVP. Moreover, of women with twin pregnancies, those for whom one or both infants were female had an increased risk of both presence of NVP and severe NVP compared to those for whom both infants were male. Fetal sex and birth plurality are factors that cannot be controlled, even if they are shown to be risk factors for NVP. However, if mothers and their partners know the fact that these factors are associated with NVP, they may be able to prepare for NVP in advance or to predict fetal sex from the severity of NVP.

Many studies on the association of HG with fetal sex or multiple births have been conducted, and most of these studies concluded that both female sex births and multiple births were associated with an increased risk for HG.^9^^–^^12^^,^^17^^–^^19^ On the other hand, conclusions drawn from these studies on the association of NVP with fetal sex have not been consistent.^6^^,^^13^^,^^20^^,^^21^ Chortatos et al showed that women who experienced NVP had higher odds of having a female infant than women with no such symptoms.^13^ Naumann et al also reported that carrying a female fetus is one of the independent predictors for NVP.^20^ Our result was comparable with these studies. However, Louik et al reported that the risk of NVP was greater for twin than for singleton births, whereas there were no substantial differences observed for the sex of the infant.^6^ Petitti also concluded that there was no significant association of nausea during pregnancy with the sex of the infant.^21^ These conflicting results may be attributed to the difference in the classification of NVP or the difference of sample size.

Human chorionic gonadotrophin (hCG) is considered to be the factor most implicated for developing NVP because both the peak of NVP and the peak of hCG production occur between 12 and 14 weeks of gestation and because NVP is often more severe in pregnant women with conditions associated with elevated hCG levels, such as molar pregnancies and multiple gestation.^1^^,^^2^ Some studies have found a positive association between serum or urinary hCG concentration and the frequency or severity of NVP, although other studies found no relationship.^22^^–^^24^ One of the most influential mechanisms underlying the higher prevalence of female offspring among mothers with NVP/HG is thought to be the differences in the concentration of hCG in the serum of pregnant women depending on the sex of fetuses. Some studies have stated that hCG concentrations were higher in the serum of women bearing female fetuses than those bearing male fetuses in mothers with singleton pregnancies.^25^^,^^26^ Steier et al showed that, among mothers pregnant with twins, significantly higher hCG concentrations in maternal blood were found only when one or both infants were female.^27^ This association between the presence of a female fetus and high hCG concentrations is similar to the association between the presence of a female fetus and presence or severity of NVP found in this study. These findings suggest that sex-related differences of hCG concentration affect the differences in the severity of NVP. However, these fetal sex-related differences in hCG concentration were only found in the third trimester, and these past studies did not succeed in finding differences in hCG concentration between women with female fetuses and those with male fetuses in the first trimester, when most cases of NVP occur. To the best of our knowledge, this is the first report that estimated interactions between fetal sex and birth plurality on NVP. Although the main effects of both fetal sex and birth plurality were significant, the interaction between them was not significant. A simple interpretation of these results is that there is a risk factor shared in both sexes but more abundant in the female sex. In this case, fetal sex and birth plurality show an additive effect on NVP. This interpretation is consistent with previous studies of hCG.^22^^,^^23^

As past studies showed, high pre-pregnancy BMI, younger age, and non-smoking were found to increase the risk of NVP.^6^ Multipara was also found to be risk factor of NVP in this study, although the result of the association between NVP and parity was inconclusive in past studies.^1^^,^^6^^,^^7^ The mechanisms underlying the relationship between NVP and these factors were not clear; however, Niebyl et al mentioned that NVP was less common in women whose placental volume was smaller.^2^^,^^28^ As they mentioned, younger age, non-smoking, high pre-pregnancy BMI, multipara, and multiple pregnancy, which were showed as risk factors for NVP in this study, are associated with larger placental volume. The association among these factors and NVP could be explained via the difference of placental volume. However, the association between fetal sex and NVP could not be explained using this hypothesis because female placental volume is generally smaller than male placental volume.^28^ On the other hand, Vandraas et al showed positive association between HG and high placental weight/birthweight ratio limited to female offspring only.^29^ Not only placental volume itself but placental weight/birthweight ratio may affect NVP.

Due to these conflicting results, mechanisms for explaining the association between fetal sex, birth plurality, and NVP remain inconclusive. Because we could assess neither placental volume nor maternal serum hCG levels in this study, we were unable to explore which factors might be causative. Further studies on the relationship between differences of hCG concentration and fetal sex in the first trimester or the relationship between NVP and placental weight or placental weight/birthweight ratio are warranted.

A major strength of our study is its large sample size, which enabled us to evaluate the effects of both fetal sex and number of fetuses on NVP. We could also divide twins into three groups according to sex combinations, which showed that women with female-female twin pregnancies had highest odds for the presence of NVP and severe NVP. Compared to the studies on the association between fetal sex and HG/NVP or studies on the association between twinning and HG/NVP, studies on the combined effect of fetal sex and twinning on NVP were scarce. Moreover, to our knowledge, our study is the first to evaluate the association of fetal sex and twinning with NVP in an Asian population.

A number of limitations of our study should be taken into consideration. Information on NVP was obtained from self-reported questionnaires. Our definition of severe NVP restricted it to participants who answered that they had experienced nausea and vomiting and could not ingest a meal, but this definition may not fully capture severity because we could not obtain information on duration or frequency of NVP. Moreover, we also could not obtain information on participants’ past history of NVP or their family history of NVP, although genetic factors are considered to play an important role in NVP.^4^

### Conclusions

We found that pregnant women carrying a female fetus or twin fetuses are at increased risk for the presence of NVP and severe NVP compared to those carrying a male or a singleton fetus. Although the mechanisms underlying the higher prevalence of female and twin births among women with severe NVP are yet to be elucidated, identifying both fetal sex and birth plurality as risk factors for NVP will help further research into complex pathogenesis of NVP.

## APPENDIX A. SUPPLEMENTARY DATA

eTable 1. Maternal characteristics across birth outcomes

eTable 2. Interaction between number of female and plurality on presence of NVP and severe NVP

## APPENDIX B.

Members of JECS as of 2017 (principal investigator, Toshihiro Kawamoto): Hirohisa Saito (Medical Support Center for JECS, National Center for Child Health and Development, Tokyo, Japan), Reiko Kishi (Hokkaido Regional Center for JECS, Hokkaido University, Sapporo, Japan), Nobuo Yaegashi (Miyagi Regional Center for JECS, Tohoku University, Sendai, Japan), Koichi Hashimoto (Fukushima Regional Center for JECS, Fukushima Medical University, Fukushima, Japan), Chisato Mori (Chiba Regional Center for JECS, Chiba University, Chiba, Japan), Shuichi Ito (Kanagawa Regional Center for JECS, Yokohama City University, Yokohama, Japan), Zentaro Yamagata (Koshin Regional Center for JECS, University of Yamanashi, Chuo, Japan), Hidekuni Inadera (Toyama Regional Center for JECS, University of Toyama, Toyama, Japan), Michihiro Kamijima (Aichi Regional Center for JECS, Nagoya City University, Nagoya, Japan), Takeo Nakayama (Kyoto Regional Center for JECS, Kyoto University, Kyoto, Japan), Hiroyasu Iso (Osaka Regional Center for JECS, Osaka University, Suita, Japan), Masayuki Shima (Hyogo Regional Center for JECS, Hyogo College of Medicine, Nishinomiya, Japan), Yasuaki Hirooka (Tottori Regional Center for JECS, Tottori University, Yonago, Japan), Narufumi Suganuma (Kochi Regional Center for JECS, Kochi University, Nankoku, Japan), Koichi Kusuhara (Fukuoka Regional Center for JECS, University of Occupational and Environmental Health, Kitakyushu, Japan), and Takahiko Katoh (South Kyushu/Okinawa Regional Center for JECS, Kumamoto University, Kumamoto, Japan).
