# Supplementary material for: Severity of Nausea and Vomiting in Singleton and Twin Pregnancies in Relation to Fetal Sex: The Japan Environment and Children’s Study (JECS)
Source: J Epidemiol. 2019 Sep 5;29(9):340–6. doi: 10.2188/jea.JE20180059 (PMC6680056; doi:10.2188/jea.JE20180059)
Supplement: Supplementary file 1 [file je-29-340-s001.pdf]

eTable 1. Maternal characteristics across birth outcomes

	Singleton, male	Singleton, female	Twin, both male	Twin, male and female	Twin, both female	*p* value	Cramer's V
	n=46,581	n=44,245	n=322	n=213	n=305		
	N (%)	N (%)	N (%)	N (%)	N (%)		
**Maternal age, years**							
<20	0,399 (0.9)	0,399 (0.9)	0,001 (0.3)	0,001 (0.5)	0,000 (0.0)	0.01	0.011
20–24	4,174 (9.0)	3,936 (8.9)	0,028 (8.7)	0,013 (6.1)	0,018 (5.9)		
25–29	12,786 (27.5)	12,192 (27.6)	0,085 (26.4)	0,047 (22.1)	0,092 (30.2)		
30–34	16,371 (35.2)	15,756 (35.6)	0,112 (34.8)	0,063 (29.6)	0,108 (35.4)		
35–39	10,688 (23.0)	9,916 (22.4)	0,081 (25.2)	0,072 (33.8)	0,070 (23.0)		
≥40	2,159 (4.6)	2,046 (4.6)	0,015 (4.7)	0,017 (8.0)	0,017 (5.6)		
Missing[a]	4	0	0	0	0		
**Pre-pregnancy BMI, kg/m^2**							
<18.5	7,594 (16.3)	7,116 (16.1)	0,052 (16.2)	0,035 (16.4)	0,049 (16.1)	0.756	
18.5–24.9	33,965 (73.0)	32,477 (73.5)	0,236 (73.3)	0,152 (71.4)	0,218 (71.5)		
≥25	4,998 (10.7)	4,617 (10.4)	0,034 (10.6)	0,026 (12.2)	0,038 (12.5)		
Missing[a]	24	35	0	0	0		
**Parity**							
Primipara	19,349 (42.6)	18,385 (42.6)	154 (48.6)	107 (51.4)	158 (52.3)	<0.001	0.016
Multipara	26,059 (57.4)	24,781 (57.4)	163 (51.4)	101 (48.6)	144 (47.7)		
Missing[a]	1173	1079	5	5	3		
**Education, years**							

≤12	16,670 (50.5)	16,057 (48.6)	106 (0.3)	82 (0.3)	97 (0.3)	0.14	
>12	29,741 (51.0)	28,003 (48.0)	214 (0.4)	130 (0.2)	207 (0.4)		
Missing[a]	170	185	2	1	1		
**Smoking during pregnancy**							
No	44,069 (95.4)	41,905 (95.5)	309 (96.3)	208 (99.1)	296 (97.7)	0.028	0.011
Yes	2,131 (4.6)	1,977 (4.5)	12 (3.7)	2 (1.0)	7 (2.3)		
Missing[a]	381	363	1	3	2		

BMI, body mass index.

Chi-squared test.

[a] Not included in percentage distribution.

eTable 2. Interaction between number of female and plurality on presence of NVP and severe NVP

	Presence of NVP		Severe NVP	
	cOR (95% CI)	aOR (95% CI)	cOR (95% CI)	aOR (95% CI)
**Fetal sex and plurality**[a]				
Number of females	1.15 (1.12–1.18)	1.15 (1.12–1.18)	1.28 (1.22–1.33)	1.28 (1.23–1.34)
Plurality	1.31 (1.12–1.53)	1.37 (1.17–1.61)	1.32 (1.04–1.66)	1.38 (1.09–1.74)
Plurality # number of female	0.97 (0.83–1.14)	0.96 (0.82–1.13)	1.12 (0.90–1.40)	1.09 (0.87–1.36)

aOR, adjusted odds ratio; CI, confidence interval; cOR, crude odds ratio; NVP, nausea and vomiting in pregnancy.

[a] Adjusted for maternal age, pre-pregnancy BMI, parity, education, and smoking during pregnancy.
